# An Engineered Gene Nanovehicle Developed for Smart Gene Therapy to Selectively Inhibit Smooth Muscle Cells: An In Vitro Study

**DOI:** 10.3390/ijms21041530

**Published:** 2020-02-24

**Authors:** Ling-Yi Cheng, Yu-Chi Wang, Ming-Hong Chen, Fu-I Tung, Kuan-Ming Chiu, Tse-Ying Liu

**Affiliations:** 1 Department of Biomedical Engineering, National Yang-Ming University, Taipei 11221, Taiwan; emmeline.ly.cheng@gmail.com (L.-Y.C.); tomato13590@gmail.com (Y.-C.W.); 2 Department of Neurosurgery, Taipei Municipal Wanfang Hospital, Taipei 11696, Taiwan; 134976@gapp.fju.edu.tw; 3 Graduate Institute of Nanomedicine and Medical Engineering, Taipei Medical University, Taipei 11031, Taiwan; 4 Department of Orthopaedic Surgery, Taipei City Hospital, Taipei 11146, Taiwan; DAG47@tpech.gov.tw; 5 Department of Health and Welfare, College of City Management, University of Taipei, Taipei 10048, Taiwan; 6 Far Eastern Memorial Hospital, Taipei 22060, Taiwan

**Keywords:** in-stent restenosis, vascular endothelium cell, smooth muscle cell, gene vehicle

## Abstract

In-stent restenosis is a serious concern for patients treated through the stenting procedure, although this can be solved using drug-eluting stents and/or drug-eluting balloon catheters. However, the chemical agents released from the drug-eluting layer for inhibiting smooth muscle cell (SMC) migration are inevitably associated with damage to vascular endothelial cell (ECs). The present in vitro study used a distinct strategy, in which a smart gene (phEGR1-PKCδ, an engineered plasmid consists of an SMC-specific promoter (human early growth response 1, *hEGR1* promoter) ligated with a gene encoding apoptosis-inducing protein (protein kinase C-delta, PKCδ) was incorporated into a novel gene vehicle (Au cluster-incorporated polyethylenimine/carboxymethyl hexanoyl chitosan, PEI-Au/CHC) to form the PEI-Au/CHC/phEGR1-PKCδ complex, which was proposed for the selective inhibition of SMC proliferation. It was found that the cell viability of SMCs receiving the PEI-Au/CHC/phEGR1-PKCδ complex under simulated inflammation conditions was significantly lower than that of the ECs receiving the same treatment. In addition, the PEI-Au/CHC/phEGR1-PKCδ complex did not demonstrate an inhibitory effect on EC proliferation and migration under simulated inflammation conditions. Finally, the PEI-Au/CHC/phEGR1-PKCδ complexes coated onto a balloon catheter used in percutaneous transluminal coronary angioplasty (PTCA) could be transferred to both the ECs and the SMC layer of Sprague Dawley (SD) rat aortas ex vivo. These preliminary in vitro results suggest that the newly developed approach proposed in the present study might be a potential treatment for reducing the incidence rate of in-stent restenosis and late thrombosis in the future.

## 1. Introduction

Coronary artery disease (CAD) is one of the most fatal human diseases, and is the deadliest disease in America. CAD is usually caused by atherosclerosis, a chronic inflammatory condition in which plaque builds up inside the arteries and slows down blood flow, an effect that can lead to heart attack, stroke, or even death [1]. Angioplasty and stenting are the most widely used treatment techniques for advanced atherosclerosis. However, the blood vessels may be blocked again due to the formation of scar tissue beneath the new healthy lining, in a process called in-stent restenosis or neointima hyperplasia. This might occur within 6 months after the initial stenting procedure. Hence, reducing the incidence rate of in-stent restenosis is very important for patients treated through the stenting procedure.

In-stent restenosis, which refers to the over-proliferation and migration of vascular smooth muscle cells (SMCs), can be significantly reduced by employing drug-eluting stents or drug-eluting balloon catheters. However, the drugs which are used to inhibit SMCs, namely chemotherapeutic agents such as paclitaxel and rapamycin, are inevitably associated with issues involving vascular endothelial cells (ECs). Uncompleted EC coverage may place patients at risk of late thrombus (a highly fatal complication) at roughly 1 year after the stenting procedure [2]. Therefore, the main challenge in the use of either drug-eluting stents or drug-eluting balloon catheters becomes how to inhibit the over-proliferation and migration of SMCs without damaging ECs (i.e., SMC-specific inhibition).

In the present study, we proposed a smart gene therapy by which the apoptosis-inducing protein was selectively generated in SMCs in vitro. To inhibit the proliferation and migration of SMCs, protein kinase C-delta (PKCδ) was chosen as an apoptosis-inducing protein (functional protein) for inhibiting SMCs, because PKCδ is a critical upstream factor leading to vascular SMC apoptosis under inflammation conditions [3]. However, it has also been reported that PKCδ might induce the apoptosis of bovine ECs in inflammation conditions [4]. In addition, PKCδ expression is cell-dependent and stimuli-dependent [5,6,7,8]. To reduce the inhibition effect of PKCδ on ECs, a smart plasmid DNA was constructed using a human early growth response 1 promoter (*hEGR1* promoter) as an SMC-specific gene switch, as *hEGR1* promoter acts as a molecular switch to selectively express PKCδ in SMCs. *hEGR1* promoter is known to be an oxidative stress-activated promoter that can be differentially activated in different cells [9,10,11]. According to the results of our preliminary test, it was found that *hEGR1* promoter could act as a molecular switch in SMCs, as it is involved in switch on in SMCs under oxidative stress. Similar behavior was not found in ECs. We hypothesize that the inhibition effect of the proposed plasmid DNA (phEGR1-PKCδ, a plasmid consists of the *hEGR1* promoter ligated with the desired sequence to express PKCδ) on SMCs would be more significant than its effect on ECs. This has not been reported before and deserves systematic examination.

The above mentioned smart gene was tested in vitro using a novel gene delivery system. Au cluster-incorporated polyethylenimine (PEI) was synthesized and then mixed with carboxymethyl hexanoyl chitosan (CHC) and the phEGR1-PKCδ plasmid to form the PEI-Au/CHC/phEGR1-PKCδ complex. First, the SMC-specific switch behavior of *hEGR1* promoter was confirmed. In addition, the in vitro toxicity and transfection efficiency of the proposed vehicle were investigated. Furthermore, the inhibition effects of the PEI-Au/CHC/phEGR1-PKCδ complex on SMCs and ECs were evaluated by PrestoBlue assay, nuclei morphology, Terminal deoxynucleotidyl transferase dUTP nick end labeling (TUNEL) assay, and migration assay. Finally, a balloon catheter used in percutaneous transluminal coronary angioplasty (PTCA) coated with a cellulose layer containing the PEI-Au/CHC/phEGR1-PKCδ complex was employed ex vivo to examine whether the gene vehicle could be delivered to the SMC layer, which confirmed that the next phase (i.e., in vivo study) of our strategy was feasible. This newly developed approach might be helpful for reducing the incidence rate of in-stent restenosis and late thrombus.

## 2. Results and Discussion

### 2.1. Functionality of the phEGR1-PKCδ Gene

**Scheme 1:**
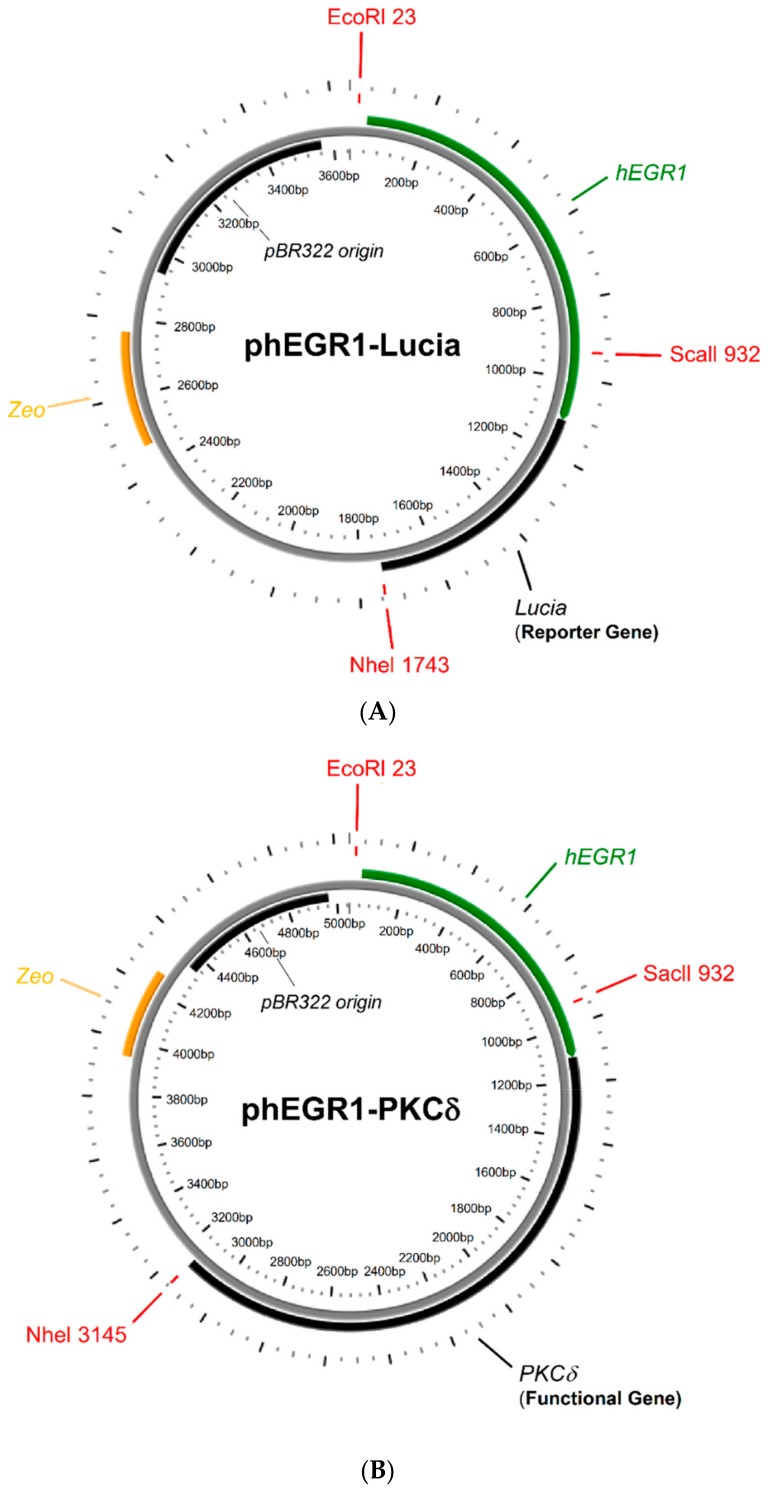
The smart genes constructed in the present study. (**A**) phEGR1-Lucia is a plasmid consisting of human early growth response 1 promoter *(hEGR1* promoter) and *Lucia* gene. (**B**) phEGR1-PKCδ is a plasmid consisting of *hEGR1* promoter and *PKCδ* gene. In this scheme, *hEFR1* promoter and *Zeo* gene were label with green and yellow, respectively. The restriction enzymes with recognition cleavage at EcoRI, SacII and NheI were label with red.

**Figure 1:**
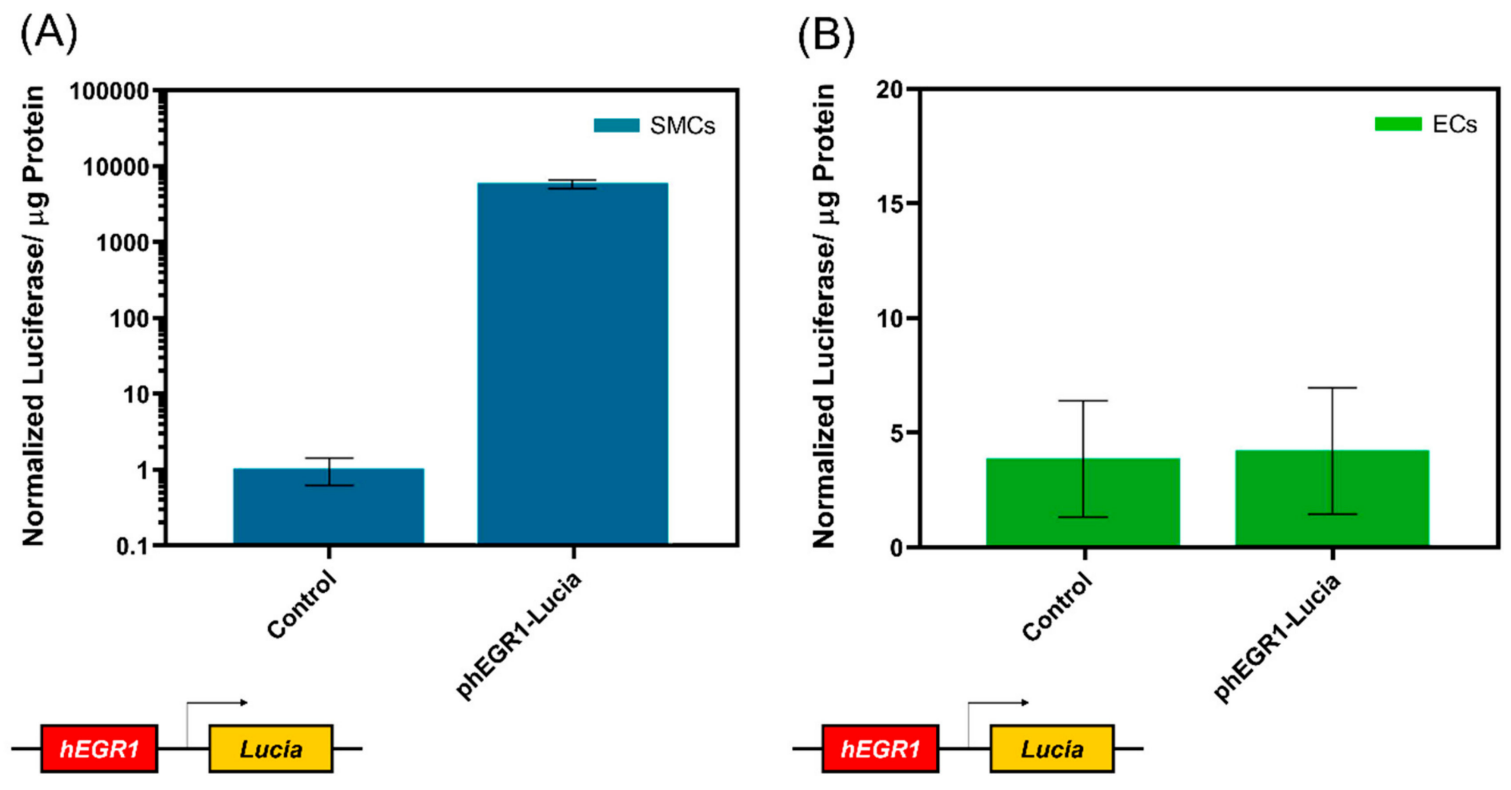
*hEGR1* promoter activities in (**A**) smooth muscle cells (SMCs) and (**B**) endothelial cells (ECs) were characterized using the *Lucia* gene as a report gene. phEGR1-Lucia was transfected by Effectene^®^ Transfection Reagent, a commercial gene vehicle, to eliminate the noise factors from vehicles.

The first objective of the present study was to construct a smart gene that specifically inhibited the proliferation and migration of SMCs without inhibiting ECs. As shown in Scheme 1, *hEGR1* promoter was respectively ligated with a reporter gene (the *Lucia* gene) and a functional gene (the *PKCδ* gene) to confirm the functionality of *hEGR1* promoter. The *Lucia* gene was used as a report gene to evaluate transfection efficiency (i.e., luciferase activity), because it is easy to precisely characterize transfection efficiency through luminescence intensity measurement. First, the phEGR1-Lucia plasmid was transfected into SMCs and ECs by using the Effectene^®^ Transfection Reagent, a commercial gene vehicle that can eliminate the noise factors during transfection, as a transfection vehicle. As shown in Figure 1A, the expression of *Lucia* in SMCs was significantly increased while *hEGR1* was employed as a promoter. On the contrary, the expression of *Lucia* in ECs was not altered while *hEGR1* was employed (Figure 1B). The results shown in Figure 1 confirm that *hEGR1* is an SMC-specific promoter.

**Figure 2:**
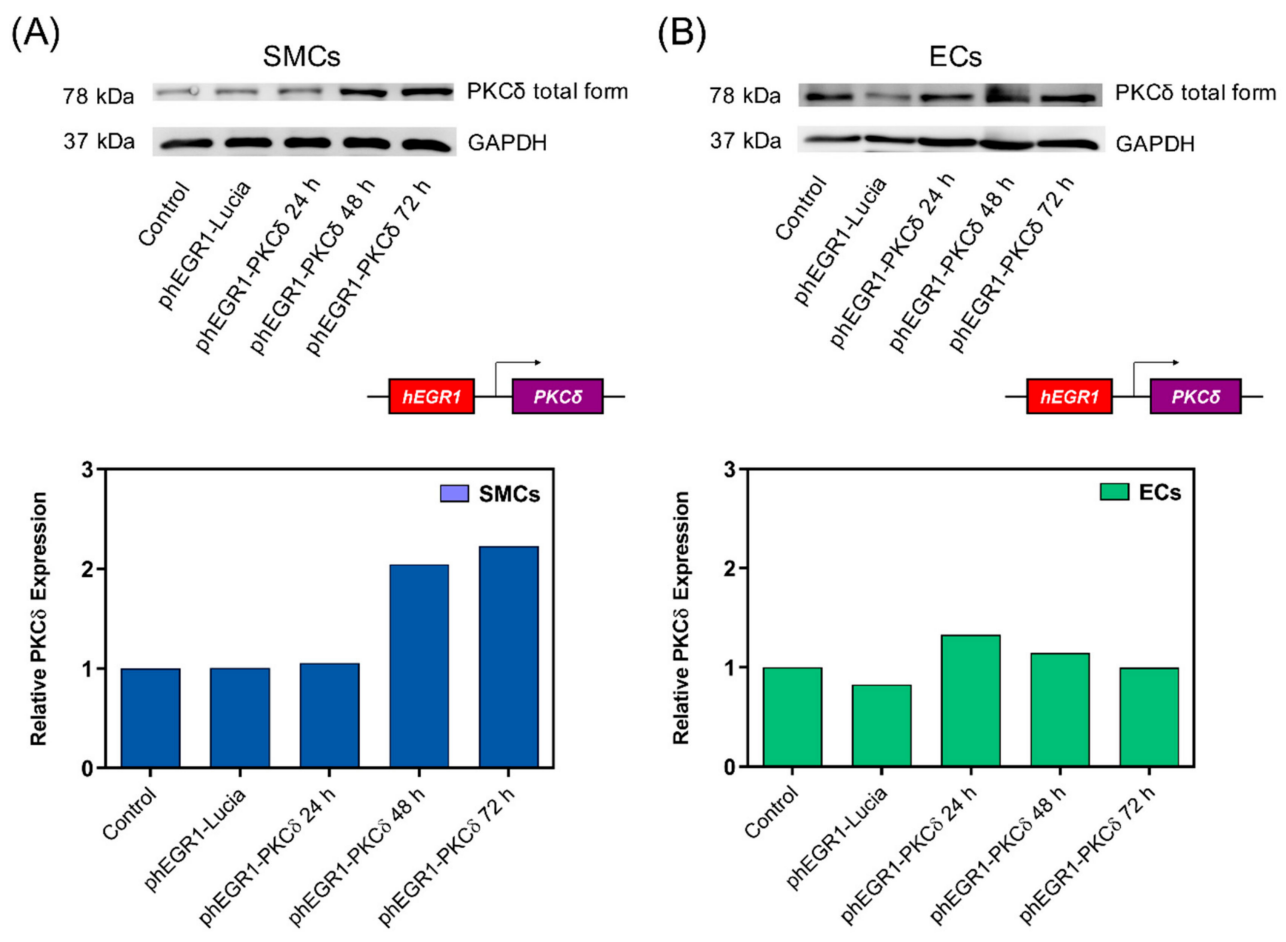
Western blot was used to evaluate the amount of functional protein (protein kinase C-delta, PKCδ) expressed in (**A**) smooth muscle cells and (**B**) endothelial cells transfected with the phEGR1-PKCδ gene using Effectene^®^ Transfection Reagent as the gene vehicle. Control groups are wild type cells which were not treated with phEGR1-Lucia or phEGR-PKCδ genes.

The SMC-specific expression of the functional protein (PKCδ) was confirmed by using a Western blot to evaluate the amount of PKCδ expressed in the SMCs and ECs transfected with the phEGR1-PKCδ gene. Wild type cells were used as control groups. As shown in Figure 2A, the expression amount of PKCδ in the phEGR1-PKCδ gene-transfected SMCs at 48 and 72 h was higher than the background level shown in the two control groups. This suggested that the functional protein was successfully produced in the phEGR1-PKCδ gene-transfected SMCs at 48 h after transfection. However, this was not observed for the ECs receiving the same treatment (Figure 2B). This implies that the first objective in the present study, the selective generation of the cell-inhibiting protein (PKCδ) in SMCs, was successfully realized via the construction of the phEGR1-PKCδ gene.

### 2.2. Characterization of Gene Vehicle (PEI-Au/CHC)

**Figure 3:**
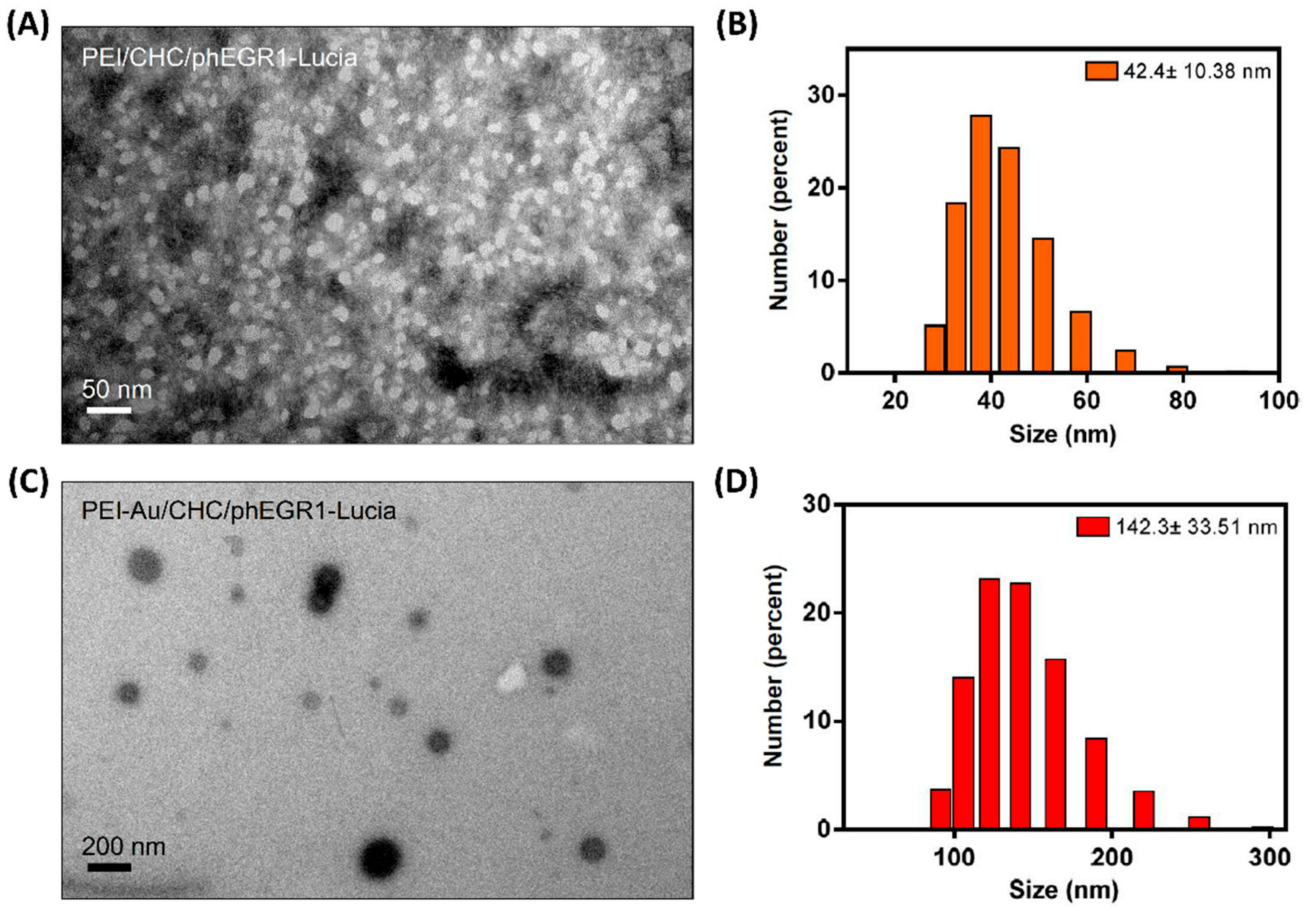
Morphology characterization of the PEI/CHC/phEGR1-Lucia (Polyethylenimine/carboxymethyl hexanoyl chitosan/phEGR1-Lucia) complex and PEI-Au/CHC/phEGR1-Lucia (Au cluster-incorporated polyethylenimine/carboxymethyl hexanoyl chitosan/phEGR1-Lucia) complex. (**A**) Transmission electron microscopy (TEM) image and (**B**) Dynamic light scattering (DLS) size distribution analysis of the PEI/CHC/phEGR1-Lucia complex. (**C**) TEM image and (**D**) DLS size distribution analysis of the PEI-Au/CHC/phEGR1-Lucia complex.

To highly exert the functionality of an engineered gene, a vehicle with biocompatibility and transfection efficiency is required. In the present study, Au cluster-incorporated polyethylenimine (PEI) was synthesized and then mixed with carboxymethyl hexanoyl chitosan (CHC) and the phEGR1-PKCδ gene to form the PEI-Au/CHC/phEGR1-PKCδ complex (i.e., a vehicle/gene complex). CHC, a water-soluble and pH-sensitive chitosan derivative with low toxicity, was employed to partially replace PEI to reduce the toxicity of the PEI-based gene vehicle. CHC molecules with negative and positive moieties could interact with PEI chains via electrostatic attraction; thus, a physically crosslinked vehicle/gene complex with lower toxicity could be prepared. However, our pilot study showed that the transfection efficiency of the CHC-containing vehicle was inevitably decreased. Therefore, Au nanoparticles were used to remedy the transfection efficiency of the CHC-containing vehicle based on the work by Thomas and Klibanov, who reported that Au nanoparticles could enhance the transfection efficiency of the PEI-based gene vehicle [12]. As shown in Figure 3, the particle sizes of the PEI/CHC/phEGR1-Lucia complex and the PEI-Au/CHC/phEGR1-Lucia complex were 42 nm and 142 nm, respectively. This implies that the particle size of the proposed gene vehicle was significantly increased after the incorporation of the Au cluster. According to our pilot tests, the PEI/DNA N/P ratio and PEI/CHC N/P ratio were important parameters for obtaining a vehicle/gene complex with compromised transfection efficiency and cell viability. The transfection efficiency (i.e., luciferase activity assay) and cell viability (i.e., PrestoBlue assay) of the PEI-Au/CHC/phEGR1-Lucia complex with different PEI/DNA ratios and PEI/CHC ratios was systemically evaluated. As shown in Figure S1A, the cells transfected with PEI-Au/DNA complexes (CHC/DNA N/P ratio = 0) with PEI/DNA ratios of 5 to 10 (columns 5 and 6 in Figure S1A) demonstrated a relatively high luminescence intensity. However, the cell viabilities of these two samples (columns 5 and 6 in Figure S1B) were relatively low, which was attributed to the toxicity of PEI. Interestingly, as shown in Figure S1B, the cell viability of PEI-Au/DNA complexes was remedied by the incorporation of CHC (CHC/DNA N/P ratios 5 to 20; shown in columns 7–21). As expected, the incorporation of CHC inevitably decreased the transfection efficiency. We compromised between cell viability and transfection efficiency, whereby the sample (i.e., column 18, indicated by the red arrow) with a PEI-Au/DNA N/P ratio at 0.5 and a CHC/DNA N/P ratio at 20 was selected to prepare the gene vehicle (zeta potential +22 mV) for delivering the smart gene constructed in the present study. It is reasonable to believe that the positively charged PEI-Au/CHC/phEGR1-PKCδ complex would tend to be tightly incorporated with the extracellular matrix containing negatively charged glycosaminoglycans (GAGs) [13]. Generally, interaction between the gene/vehicle complex and GAGs might cause a new GAGs-containing complex or release of DNA, which might affect the transfer and transfection of gene [13]. As shown in Figure S2, DNA molecules did not migrate outside of the loading wells of the hyaluronic acid (HAc)-treated and dextran sulfate-treated gene/vehicle complexes, suggesting that HAc and dextran sulfate did not cause the release of DNA. In addition, HAc-gene/vehicle complex aggregation was not observed. Light aggregation of the dextran sulfate-gene/vehicle complex was observed, implying that dextran sulfate might condense the proposed gene/vehicle complex. On the other hand, serum also possibly demonstrates condensation ability for the gene/vehicle complex. In our pilot study, a fetal bovine serum (FBS)-containing culture medium demonstrated a condensation ability for the proposed PEI-Au/CHC/phEGR1-PKCδ complex. The interaction between the gene/vehicle and polyanions in vivo is an important issue for gene vehicles; however, controversy still exists [14,15,16,17,18,19]. It may not be easy to mimic in vivo gene transfer in real GAGs-rich matrix via in vitro methodology. We need more in vivo research to systemically explore the transfer, transfection efficiency and therapeutic efficacy of the proposed gene vehicle.

**Figure 4:**
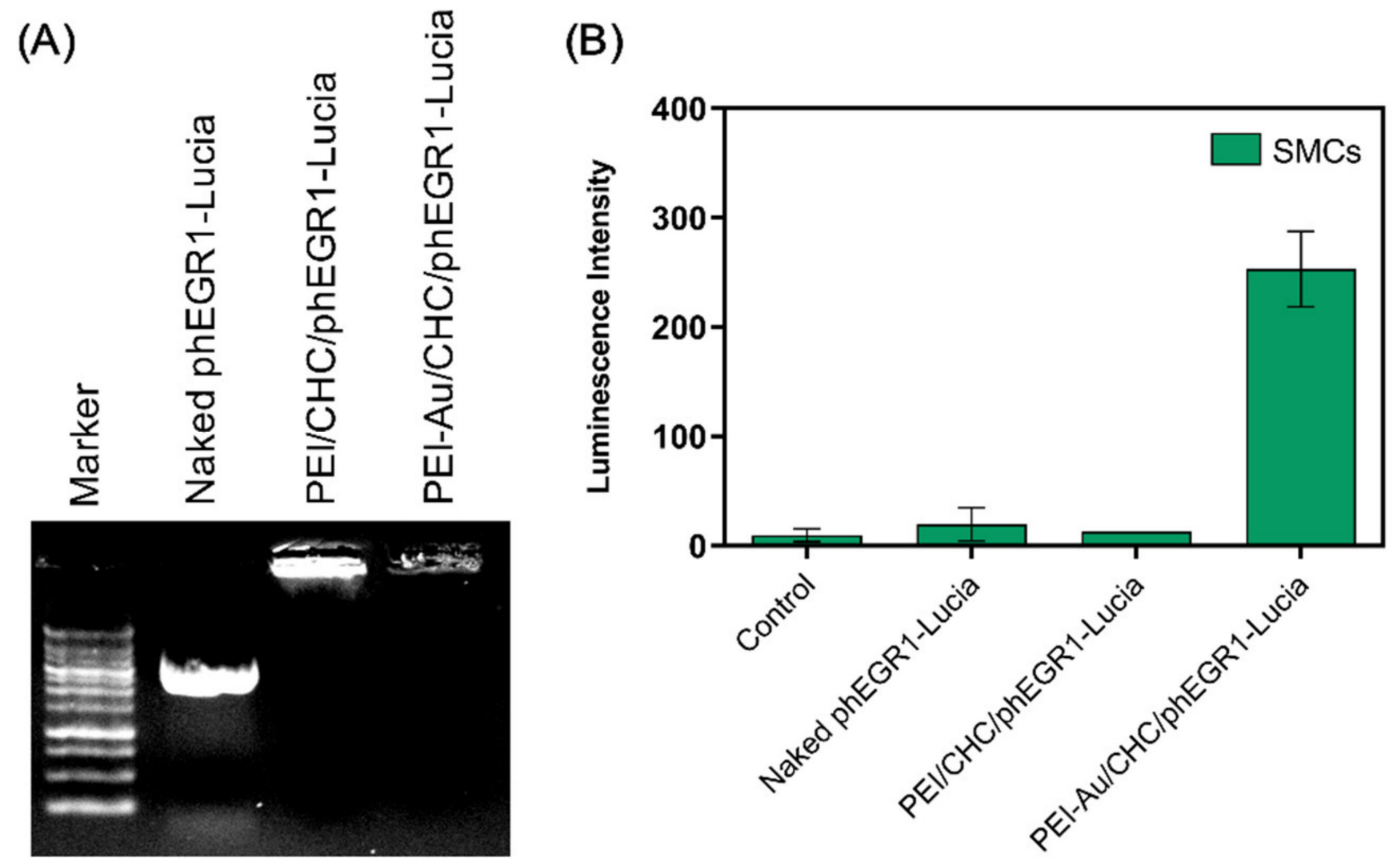
(**A**) The encapsulation ability of the polyethylenimine/carboxymethyl hexanoyl chitosan (PEI/CHC) and Au cluster-incorporated polyethylenimine/carboxymethyl hexanoyl chitosan (PEI-Au/CHC) vehicles for the phEGR1-Lucia gene. The condensation effects of the PEI/CHC vehicle and PEI-Au/CHC vehicle on DNA were investigated using electrophoresis on 1.2% agarose gel with Tris-acetate (TAE) running buffer at 80 V for 25 min. DNA was visualized with EtB “Out” nucleic acid staining solution. (**B**) In vitro transfection efficiency of the PEI/CHC/phEGR1-Lucia complex and PEI-Au/CHC/phEGR1-Lucia complex.

The effect of Au on the proposed vehicle was also investigated. First, the effect of Au on the gene encapsulation was examined using a condensation test which was performed via electrophoresis on 1.2% agarose gel with Tris-acetate (TAE) running buffer at 80 V for 25 min. DNA was visualized with EtB “Out” nucleic acid staining solution. As shown in Figure 4A, bright band (stained DNA) was not observed outside of the loading wells of the PEI/CHC/phEGR1-Lucia complex and PEI-Au/CHC/phEGR1-Lucia complex, suggesting that DNA did not migrate outside of their loading wells [20]. This implies that both PEI/CHC and PEI-Au/CHC vehicles demonstrated a good capability to encapsulate the genes. This might be attributed to the crosslinked structure of the vehicle/gene complex mentioned before. In this inorganic–organic structure, the Au cluster exerted a shield effect for the optical signal emitted from EtB “Out”-stained DNA. Hence, the optical intensity in the third loading well was significantly lower than that in the first and second loading wells. Subsequently, in vitro transfection efficiencies of the PEI/CHC/phEGR1-Lucia complex and PEI-Au/CHC/phEGR1-Lucia complex were characterized by using *Lucia* as a reporter gene. As shown in Figure 4B, it was found that the transfection efficiency of PEI-Au/CHC vehicle was higher than that of the PEI/CHC vehicle. This finding is in accordance with the observation of Thomas and Klibanov’s report. In addition, this can probably be attributed to the size-dependent internalization, as shown in Figure 3.

### 2.3. Efficacy of PEI-Au/CHC/phEGR1-PKCδ Complex

**Figure 5:**
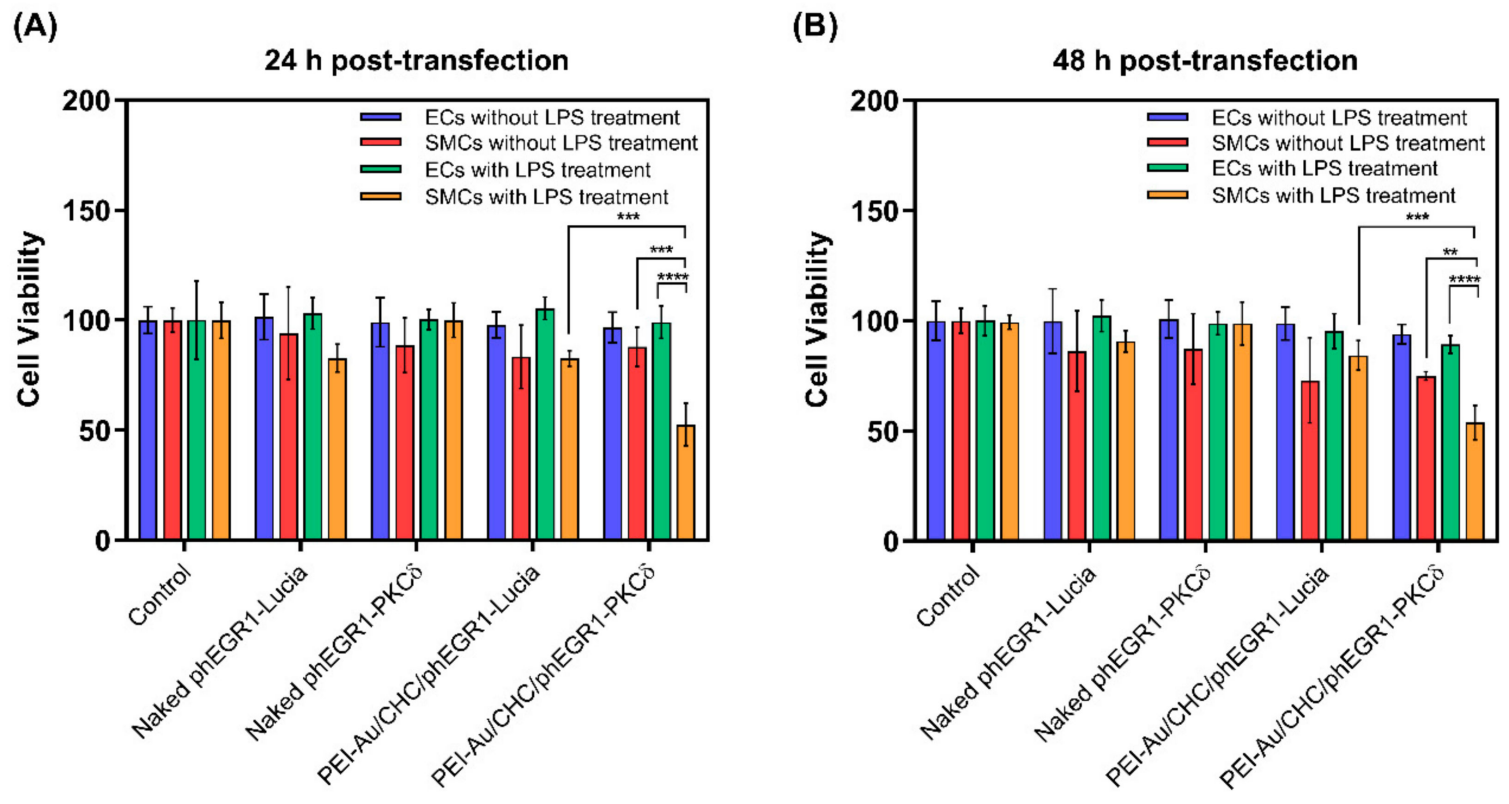
The in vitro inhibition effect of the PEI-Au/CHC/phEGR1-PKCδ complex on the smooth muscle cells and endothelial cells was evaluated with PrestoBlue^®^ reagent at (**A**) 24 h and (**B**) 48 h after transfection. The cell viability of endothelial cells and smooth muscle cells treated with differing PEI-Au/CHC/DNA complexes was assessed. Cells were treated with lipopolysaccharides (LPS) to simulate the inflammatory environment. ** *p* ≤ 0.01, *** *p* ≤ 0.001, **** *p* ≤ 0.0001.

After the confirmation of transfection efficiency and biocompatibility of the proposed gene vehicle (PEI-Au/CHC), it was employed to deliver the phEGR1-PKCδ gene into SMCs and ECs. In vitro, the inhibition effect of PEI-Au/CHC/phEGR1-PKCδ complex on the cell viability of SMCs and ECs was evaluated via using PrestoBlue^®^ reagent at 48 h after transfection. Lipopolysaccharides (LPS) was used to simulate the inflammatory condition. As can be seen in Figure 5, it was found that the cell viability of SMCs receiving the PEI-Au/CHC/phEGR1-PKCδ complex was significantly lower than that of ECs receiving the same treatment under inflammation simulation conditions. This implies that the selective inhibition effect on SMC proliferation was realized via the PEI-Au/CHC/phEGR1-PKCδ complex. Interestingly, as can be seen, the phEGR1-PKCδ gene could not exert its functionality on inhibiting SMC without employing PEI-Au/CHC as a gene vehicle. This is in accordance with the results shown in Figure 4. The results suggested that PEI-Au/CHC had a key role in exerting the functionality of the phEGR1-PKCδ gene, which was probably attributed to the vehicle-enhanced internalization. Most importantly, the Au nanoparticle has been reported as an endoplasmic reticulum (ER) stress enhancer that can translocate PKCδ into the ER and then activate PKCδ by Ab1 (i.e., one kind of tyrosine kinase). The resulting complex would be transported to mitochondria, leading to the mitochondrial apoptosis pathway. On the other hand, PKCδ could be activated by LPS stimulation, which also induces the mitochondrial apoptosis pathway. These two mitochondria apoptosis pathways may be the cause of the enhancement of the selective inhibition effect of the PEI-Au/CHC/phEGR1-PKCδ complex on SMCs under the LPS stimulation condition [21,22].

**Figure 6:**
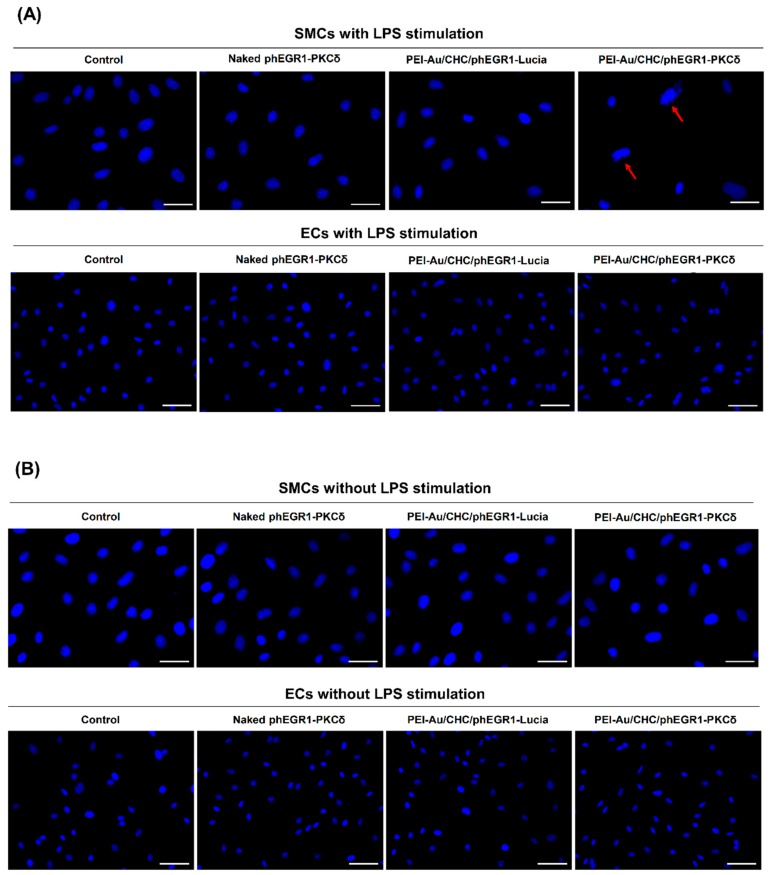
Nuclei morphology of ECs and SMCs treated with naked phEGR1-PKCδ, the PEI-Au/CHC/phEGR1-Lucia complex, and the PEI-Au/CHC/phEGR1-PKCδ complex (**A**) with LPS and (**B**) without LPS stimulation. All cells were stained with 4’,6-diamidino-2-phenylindole (DAPI) to label cell nucleus (blue). Arrow marks indicate apoptotic nuclei. Scale bar, 50 μm.

**Figure 7:**
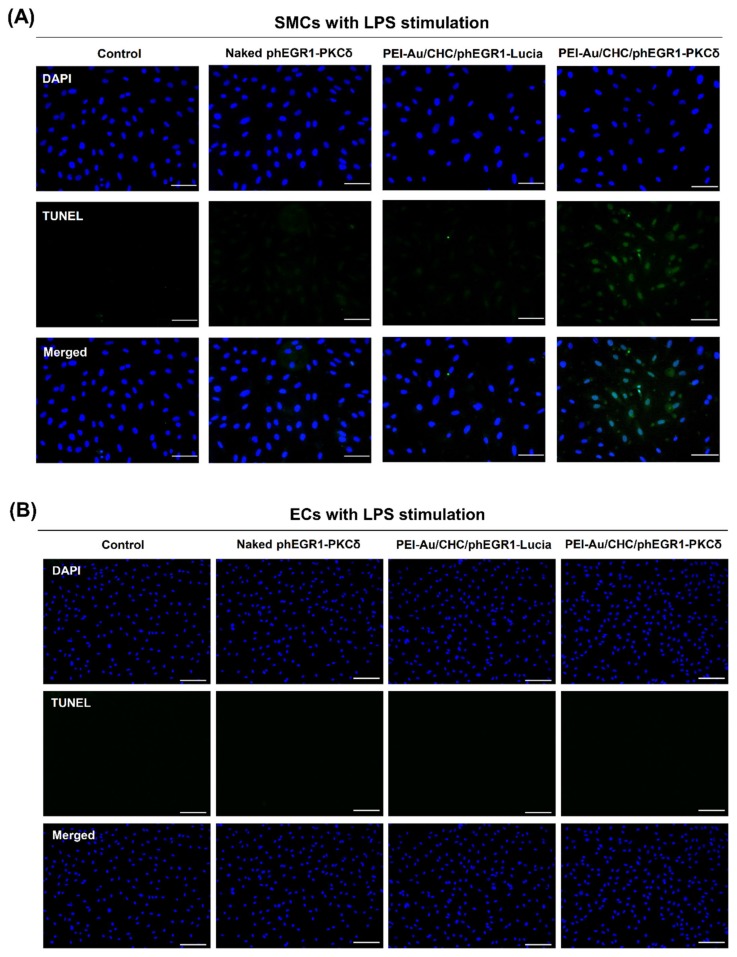

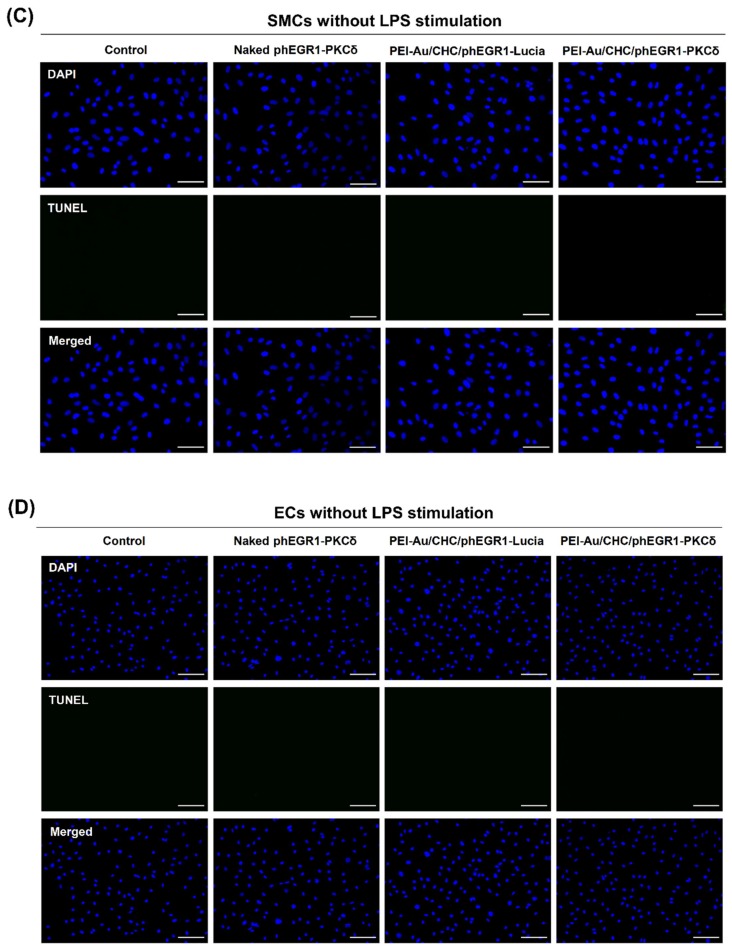
Terminal deoxynucleotidyl transferase dUTP nick end labeling (TUNEL) assay of (**A**) SMCs and (**B**) ECs treated with naked phEGR1-PKCδ, the PEI-Au/CHC/phEGR1-Lucia complex, and the PEI-Au/CHC/phEGR1-PKCδ complex under conditions with LPS stimulation. TUNEL assay of (**C**) SMCs and (**D**) ECs treated with naked phEGR1-PKCδ, the PEI-Au/CHC/phEGR1-Lucia complex, and the PEI-Au/CHC/phEGR1-PKCδ complex under conditions without LPS stimulation. All cells were stained with TUNEL (green) to label DNA fragmentation and DAPI (blue) to label cell nucleus. Scale bar, 100 μm.

The PrestoBlue assay suggested that the SMC-specific inhibition of proliferation under inflammation condition was realized via using the PEI-Au/CHC/phEGR1-PKCδ complex. This was further supported by nuclei morphology and the TUNEL assay. As shown in Figure 6, ruptured nuclei (indicated by red arrows) were clearly observed for the SMCs treated with the PEI-Au/CHC/phEGR1-PKCδ complex under simulated inflammation conditions. This was not observed for the ECs treated with the same conditions. In addition, ruptured nuclei were not observed for the cells treated with the naked phEGR1-PKCδ gene, supporting the functionality of the PEI-Au/CHC vehicle. Importantly, the PEI-Au/CHC/phEGR1-PKCδ complex did not exert considerable inhibiting efficacy without the inflammation condition, implying that the inhibiting efficacy of our approach would not be triggered in healthy SMCs. The above observation of nuclei morphology is in accordance with the results of the TUNEL labeling test. As can be seen in Figure 7, TUNEL-labeled cells (green), which indicate the presence of DNA fragments, were only found in PEI-Au/CHC/phEGR1-PKCδ complex-treated SMCs under simulated inflammation conditions. This was not found in PEI-Au/CHC/phEGR1-PKCδ complex-transfected ECs with or without treatment with the inflammation condition. In summary, according to the results shown in Figure 6 and Figure 7, it is believed that our approach could exert a selective inhibiting efficacy for SMC proliferation under inflammation conditions. In other words, the inhibiting effect would not be triggered in healthy SMCs and ECs.

**Figure 8:**
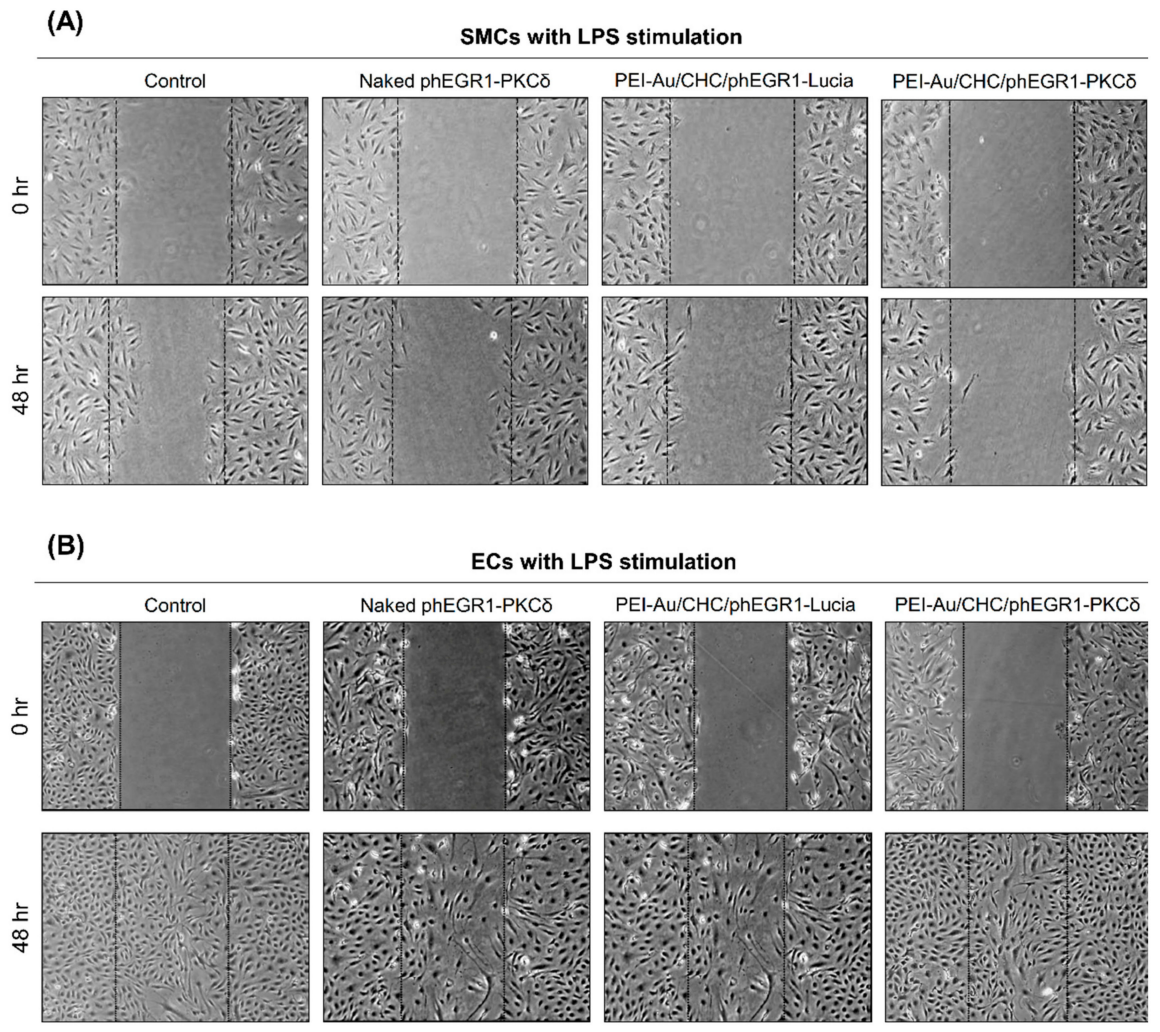
Cell migration assay performed on the serum-starved (**A**) SMCs and (**B**) ECs transfected with naked phEGR1-PKCδ, the PEI-Au/CHC/phEGR1-Lucia complex, and the PEI-Au/CHC/phEGR1-PKCδ complex under the conditions with LPS stimulation. Cell migration was measured after 48 h.

In Figure 5, Figure 6 and Figure 7, it is shown that ECs and SMCs were not significantly altered under inflammation conditions. Interestingly, we conclude that the proposed PEI-Au/CHC/phEGR1-PKCδ complex would be differently activated in SMCs and ECs under inflammation conditions. Under the same inflammation condition, the effect of the proposed PEI-Au/CHC/phEGR1-PKCδ complex on cell mobility was investigated via a migration assay. As can be seen in Figure 8, the mobility of SMCs in each group under simulated inflammation conditions was very low, which might be attributed to the effect of serum-free culture. On the other hand, the migration ability of ECs was not affected by the proposed approach, implying that the PEI-Au/CHC/phEGR1-PKCδ complex did not inhibit EC migration under the inflammation condition. It is expected that our approach is good for repairing the inner surface of blood vessel and reducing the risk of acute thrombus and late thrombus.

**Figure 9:**
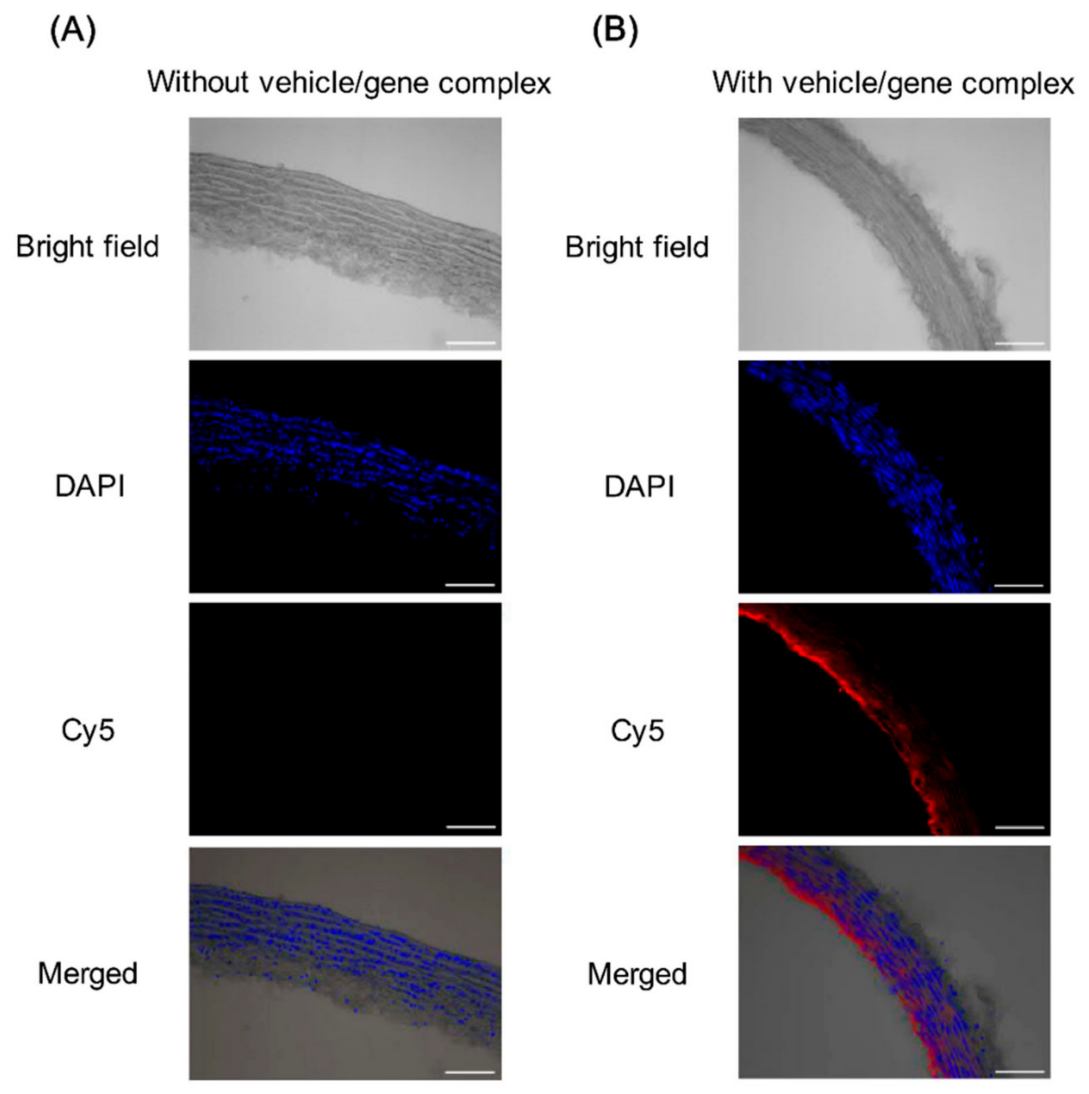
Coating layers (**A**) without and (**B**) with the PEI-Au/CHC/phEGR1-PKCδ complex were coated onto the surface of the percutaneous transluminal coronary angioplasty (PTCA) balloon catheters. phEGR1-PKCδ was stained by Cy5 (a kind of DNA stain). The DNA-incorporated balloon catheters were then attached the inner surface of blood vessels, at a scale bar of 100 μm.

To confirm the feasibility of the proposed strategy for the next phase (in vivo study), a cellulose-based matrix layer containing the PEI-Au/CHC/phEGR1-PKCδ complex was coated onto the surface of a PTCA balloon catheter, which is a common medical device used for patients suffering from atherosclerosis. The DNA incorporated was stained with a fluorescent dye (Cy5) before the formation of the vehicle/gene complex. The PTCA balloon catheter, including the Cy5-stained DNA, was then attached to the inner surface of the aorta of Sprague Dawley (SD) rats ex vivo. As shown in Figure 9, the Cy5-stained DNA could be observed in SMC layers, which implies that the PEI-Au/CHC/phEGR1-PKCδ complex can be delivered to SMCs by the PTCA balloon catheter ex vivo.

This report is a preliminary (i.e., in vitro) study to confirm the selective inhibiting efficacy of the PEI-Au/CHC/phEGR1-PKCδ complex on the proliferation and migration of inflamed SMCs without an accompanying impact on healthy SMCs and ECs. Importantly, we did not use any chemotherapeutic agents. Instead, we used gene therapy together with a local delivery route to specifically generate an intrinsic protein for inhibiting the inflamed SMCs without an accompanied impact on healthy cells. In the present study, the PEI-Au/CHC/phEGR1-PKCδ complex was delivered by the PTCA balloon catheter rather than by the intravenous route, a method through which the risk of the proposed materials might be reduced. It is known that each therapy is inevitably accompanied by risk. Therefore, we have conducted some in vivo tests with respect to nanoparticle-induced vascular problems (i.e., acute thrombus and late thrombus) and the efficacy of in vivo transfection. In addition, assessment of the reactive oxygen species (ROS) level of SMCs and ECs under the inflammation condition in the in vivo model is very complicated because it can be altered by the glucose level, extent of hypoxia, and inflammatory cytokines. This alteration might in turn affect the SMC-specific expression of functional genes in the in vivo model. Through the completed in vivo studies and further extensive exploration, the safety and efficacy of the newly developed approach will be understood and published separately in the near future.

## 3. Materials and Methods

### 3.1. Construction of phEGR1-PKCδ

#### 3.1.1. Recombinant Plasmid DNA

The phEGR1-Lucia gene and phEGR1-PKCδ gene were constructed as shown in Scheme 1. The plasmid DNA (phEGR1-PKCδ and phEGR1-Lucia) and the promoter (*hEGRI*) used in the present study were obtained by the digestion process using restriction enzymes with recognition cleavage at SacII and NheI, respectively. The reaction configuration of enzyme cleavage is shown in Table 1. Vector and insert DNA were subjected to ligation reactions with contents as shown in Table 2. The reaction was carried out overnight at 4 °C.

#### 3.1.2. Bacterial Culture and DNA Amplification

*Escherichia coli* strain DH5α was cultured on an Luria-Bertani (LB) agar plate or LB broth with the corresponding antibiotics. Bacteria were grown at 37 °C for 16 h while shaking at 250 rpm. A small piece of plasmid DNA was taken and placed into the LB broth with the corresponding antibiotic and was cultured overnight. The bacteria were collected under high-speed centrifugation and then the DNA was obtained using the HiYield Plasmid Mini Kit (Arrowtec, Taiwan).

#### 3.1.3. DNA Extraction and DNA Retardation Assay

Plasmid DNA-embedded bacteria were cultured in LB broth for one day and further collected for extraction. Plasmid DNA extraction was performed with the HiYield Plasmid Mini Kit (Arrowtec, Taiwan). Experimental steps, except for altering of final elite volume to 100 μL, are listed in the user manual. The naked DNA and vector/DNA group were prepared with loading dye addition. Then, 1.2% agarose gel with 0.5× Tris-acetate-EDTA (TAE) buffer and pre-staining with EtB “Out” Nucleic Acid Staining Solution were prepared. The gel was run with an 80-V setting for 20 min. The resulting photograph was taken with a GE LAS-4000 luminescence imaging system.

### 3.2. Measurement of Protein Production

Dulbecco’s Modified Eagle Medium (DMEM) medium, Dulbecco’s PBS, 0.25% trypsin and DAPI were purchased from Invitrogen. Rat thoracic aorta smooth muscle cells (A10) were obtained from Food Industry Research and Development Institute, Taiwan. Rat aortic endothelial cells were purched from Angio-Proteomie, USA. Smooth muscle cell strain A10 was cultured in DMEM with 10% fetal bovine serum (FBS) and 1% penicillin streptomycin (PS), incubated at 37 °C, under 5% CO_2_. The medium was renewed every 2~3 days. The endothelial cell strain was cultured in endothelial growth medium, incubated at 37 °C, with 5% CO_2_.

*hEGR1* promoter activities in smooth muscle cells and endothelial cells were characterized using *Lucia* as a report gene. phEGR1-Lucia was transfected by using Effectene^®^ Transfection Reagent (QIAGEN), a commercial gene vehicle, to eliminate the noise factors from vehicles. *Lucia* expression was characterized via using the in vivo imaging system (IVIS; PerkinElmer). Western blot was used to evaluate the amount of functional protein (PKCδ) expressed in the smooth muscle cells and endothelial cells transfected with phEGR1-PKCδ using Effectene^®^ Transfection Reagent (QIAGEN) as the gene vehicle. Control groups were wild type cells. Cells were cultured in a 6-cm culture dish and then transfected with 3000 ng/dish DNA. The transfection protocol is described in the Effectene^®^ Transfection Reagent handbook. Protein was collected at 24, 48, and 72 h after transfection.

### 3.3. Preparation of the Gene Vehicle

Hydrogen tetrachloroaurate(III) trihydrate (99.99%) was purched from Alfa Aesar. Polyethylenimine, branched (MW~25 kDa), chitosan (MW 50-190 kDa) and chloroacetic acid were purched from Sigma-Aldrich.

#### 3.3.1. Preparation of CHC

In this stage, 5 g of chitosan powder were suspended in isopropanol for 30 min. This suspension was then mixed with 12.5 mL of NaOH solution (15 N). After that, 37.5 g of chloroacetic acid were added into the resulting mixture and stirred for 30 min. The mixture was then kept at 60 °C and stirred for 4 h. After cooling down, the solution was filtrated using 1:9 water–methanol solution. The obtained product (2 g) was then dissolved in 100 mL distilled water and stirred with 100 mL methanol for one day, followed by the addition of 2.8 mL hexanoyl anhydride and stirring for 16 h. The end product was then dialyzed and dried for incubation at 50 °C.

#### 3.3.2. In-Situ Synthesis of PEI-Au Nanoparticles

Seventy-five milligrams of branched PEI were dissolved in 1.17 mL ethanol under vortex. Then, 100 μL of HAuCl_4_ solution (10 mM) were added drop by drop into the PEI solution and further stirred for 6 h until the color of the solution turned to pale yellow. The solution was stored at −20 °C.

#### 3.3.3. Quantitative Characterization of the Amine Group for the Gene Vehicles

Mixture A was prepared by dissolving 0.5 g of ninhydrin into 20 mL of ethylene glycol monomethylether. Mixture B was prepared by mixing 0.2 g of tin chloride with 10 mL of NaOH solution (1.0 M), followed by the addition of deionized water to keep the total volume at 20 mL. Mixture A was prepared with the same volume as mixture B, and then 1 mL of the resulting mixture was mixed with a 200 μL sample. This was heated at 100 °C for 20 min and then diluted (3-fold) before examining the absorbance at 570 nm. The calibration curve was plotted using standard samples obtained by the serial dilution of a glycin solution (5 micronmole/mL).

#### 3.3.4. Assembling the Nanocomplex

Different concentrations of PEI-Au, DNA, and CHC solutions were mixed to prepare vehicle/gene complexes with differing N/P ratios.

#### 3.3.5. Vehicle Characterization

The morphology and size of the prepared PEI-Au nanoparticles were examined by performing TEM. The mean diameter of the drug vehicles was measured using DLS (Malvern, ZS90).

### 3.4. Plasmid DNA Transfection

Once the cultured cells had reached 70% confluence, the nanocomplex was co-cultured with an FBS- and antibiotic-deprived medium. For this, 200 ng DNA/well (96 well plate) were used in the transfection experiments. In addition, 200 ng/mL LPS were used in inflammation simulation groups. LPS is an inflammatory stimulus. By activating up-stream proteins such as toll-like receptor 4, cells will produce proinflammatory cytokines such as interleukin (IL)-8. We therefore used the LPS co-culture to simulate the inflammatory micro-environment in which stenosis occurs [23,24,25,26].

### 3.5. The Efficacy of PEI-Au/CHC/phEGR1-PKCδ Complex

#### 3.5.1. Cell Viability

The in vitro inhibition effects of the PEI-Au/CHC/phEGR1-PKCδ complex on the smooth muscle cells and endothelial cells were evaluated with PrestoBlue^®^ reagent (Life technologies, Thermo Scientific, USA) at 24 h and 48 h after transfection. The cell viability of endothelial cells and smooth muscle cells treated with differing PEI-Au/CHC/DNA complexes was assessed. Cells were treated with LPS to simulate the inflammatory environment. Then, 24 and 48 h post-transfection, PrestoBlue^®^ reagent diluted 20-fold with FBS and antibiotic-deprived medium was used to examine cell viability. For this, 100 μL reagent were added to each well, and the trays allowed to stand for 2 h. After transferring the media to a black 96-well plate, the TECAN Sunrise ELISA Reader was used to measure fluorescence intensity. The excitation/emission wavelength was set to 560/590.

#### 3.5.2. ECs and SMC Nuclei Morphology

After 24 h of transfection, cells were washed with PBS, followed by the staining process. Then, all cells were stained with DAPI and observed by fluorescence microscopy (DM 6000B, Leica, Germany) [27].

#### 3.5.3. TUNEL Assay

After 24 h of transfection, cells were washed with PBS, followed by the staining process. The staining process is listed in the ApoAlert^TM^ DNA Fragmentation Assay Kit handbook (Clontech, USA). TUNEL-positive cells emit green light. 4′,6-Diamidino-2-phenylindole (DAPI) was used to stain cell nuclei. Images were captured with fluorescence microscopy (DM 6000B, Leica, Germany) [28].

#### 3.5.4. Wound Healing Assay

Cells were cultured in 6-well plates until the confluence reached 100%. After transfection with the nanocomplex, wounds were created with a 1 mL pipette tip and set as 0 h. Cells were incubated with FBS- and PS-deprived medium for 48 h. Pictures of wounds were taken with a microscope camera [29].

### 3.6. The Transfer Effect of the PTCA Balloon Catheter with Gene Vehicle

Sprague Dawley (SD) rats (6–8 weeks old) were purchased from the Laboratory Animal Center of National Yang-Ming University. All animals used in our experiments were treated and housed following a protocol approved by the Institutional Animal Care and Use Committee of National Yang-Ming University. To confirm the transfer effect of PEI-Au/CHC/hEGR1-PKCδ/Cy5 on the PTCA balloon catheter, ex vivo experiments were implemented. First, we prepared the PEI-Au/CHC/phEGR1-PKCδ complex according to the method above, wherein we stained DNA by using Cy5 (i.e., fluorescent dye, Thermofisher Scientific.) Then, we withdrew an arterial blood vessel from an SD rat and inserted a PTCA balloon catheter with the PEI-Au/CHC/hEGR1-PKCδ/Cy5 gene complex into the rat’s aorta. After the PTCA balloon catheter was attached the rat’s aorta, the tissue was embedded with optimal cutting temperature (OCT) gel for frozen sections. Finally, the frozen sections were observed under a fluorescence microscope (nuclei were stained with DAPI).

### 3.7. Statistical Analysis

Prism (Version 6, GraphPad Software, Inc., CA, USA) was used to analyze the data. Data in graphs were presented as mean ± S.D. Two groups of data were analyzed with unpaired *t*-tests. Comparison analysis was considered statistically significant if *p* < 0.05.

## 4. Conclusions

In the present study, a novel smart gene, phEGR1-PKCδ, was successfully constructed to exert the SMC-specific expression of an apoptosis-inducing protein (PKCδ). In addition, the smart gene was incorporated into the novel gene vehicle PEI-Au/CHC, with satisfactory toxicity and transfection efficiency because of the incorporation of CHC and Au. The PEI-Au/CHC/phEGR1-PKCδ complex showed significant inhibition efficacy with respect to the proliferation and migration of inflamed SMCs, without an accompanied impact on healthy SMCs and ECs. Furthermore, the PEI-Au/CHC/phEGR1-PKCδ complex could be delivered to the SMC layer of the aorta of SD rats using a PTCA balloon catheter coated with a cellulose-based matrix layer containing the PEI-Au/CHC/phEGR1-PKCδ complex.

## Figures and Tables

**Table 1:** Restriction enzyme cleavage reaction configuration.

Items	Amount
DNA for digestion	1 μg
Restriction enzyme 1	1 U
Restriction enzyme 2	1 U
10× cut-smart buffer	5 μL
Addition of diH_2_O to total volume	50 μL

**Table 2:** Ligation response configuration.

Items	Amount
Vector DNA	100 ng
Insert DNA	17 ng
10× ligase buffer	1 μL
T4 DNA ligase	1 U
Addition of diH_2_O to total volume	10 μL

## Supplementary Materials

Supplementary materials can be found at https://www.mdpi.com/1422-0067/21/4/1530/s1.

## Abbreviations

SMC	Smooth muscle cell
EC	Endothelial cell
PKCδ	Protein kinase C-delta
p*hEGR1*	*hEGR1* promoter
PTCA	Percutaneous transluminal coronary angioplasty
SD	Sprague Dawley
CAD	Coronary artery disease
PEI	Polyethylenimine
CHC	Carboxymethyl hexanoyl chitosan
FBS	Fetal bovine serum
PS	Penicillin streptomycin
GAGs	Glycosaminoglycans
HAc	Hyaluronic acid
TEM	Transmission electron microscopy
TUNEL	Terminal deoxynucleotidyl transferase dUTP nick end labeling
ROS	Reactive oxygen species
LPS	Lipopolysaccharides
DLS	Dynamic light scatter
IL	Interleukin
LB	Luria-Bertani
TAE	Tris-acetate-EDTA
OCT	Optimal cutting temperature
DMEM	Dulbecco’s Modified Eagle Medium
