# Supplementary material for: An Engineered Gene Nanovehicle Developed for Smart Gene Therapy to Selectively Inhibit Smooth Muscle Cells: An In Vitro Study
Source: Int J Mol Sci. 2020 Feb 24;21(4):1530. doi: 10.3390/ijms21041530 (PMC7073206; doi:10.3390/ijms21041530)
Supplement: Supplementary file 1 [file ijms-21-01530-s001.zip › supplementary.docx]

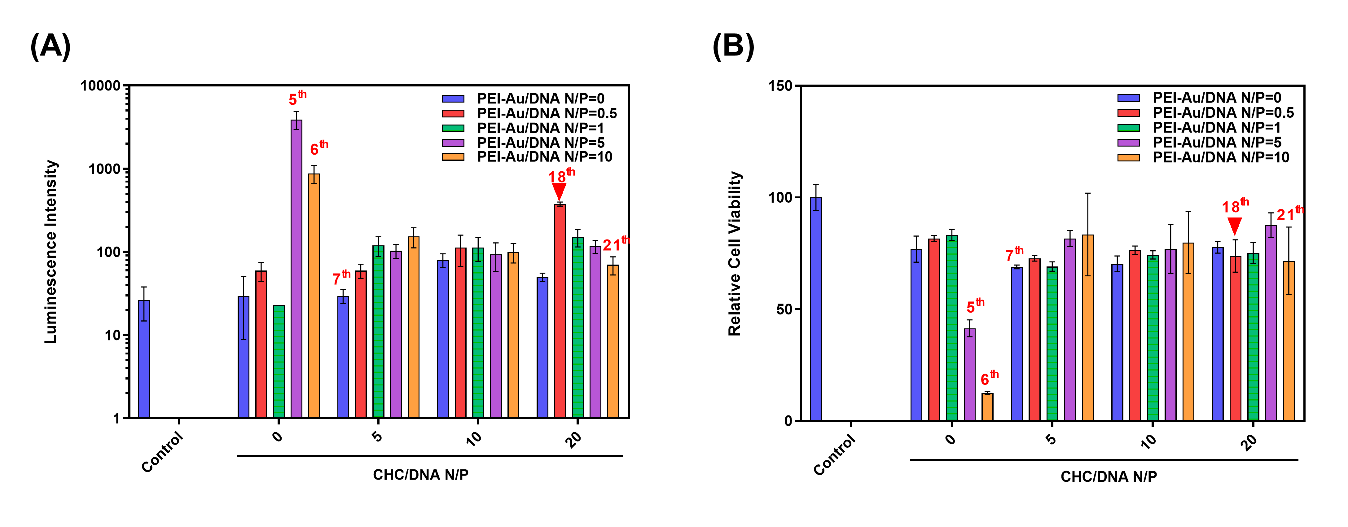


**Figure 1.** The (A) transfection efficiency (i.e., Luciferase activity) and (B) cell viability of PEI-Au/CHC/DNA complex with differing CHC/DNA ratios and PEI-Au/DNA ratios were systemically evaluated. Transfection was performed at a dose of 100 ng of phEGR1-Lucia plasmid for all groups. Cells were incubated in the transfection solution for 12 h and then changed to growth medium for 18 h before the luciferase assay and cell viability assay. CHC/DNA N/P ratio at 20 and PEI-Au/DNA N/P ratio at 0.5 (red arrow) was chosen because it demonstrated satisfied transfection efficiency and cell viability. It was found that the toxicity of PEI was successfully remedied by the incorporation of CHC. Here, DNA means phEGR1-Lucia. Each error bar was the measurement error obtained in a single experiment.


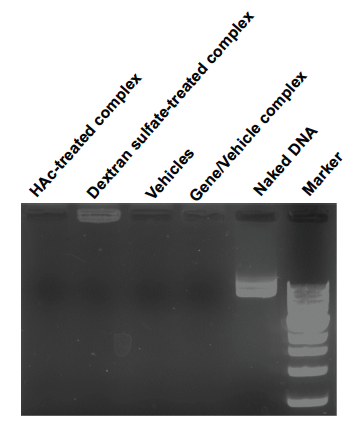


**Figure 2.** Polyplexes susceptibility test of the proposed gene/vehicle complex (i.e., PEI-Au/CHC/phEGR1-PKCδ complex) under the hyaluronic acid (HAc)-containing condition and dextran sulfate-containing condition (i.e., Dextran sulfate can be considered as a model polyanion for GAGs). The proposed gene/vehicle complex was respectively mixed with HAc and dextran sulfate. The negative/positive charged ratio was 3 and treating time was 4 hours. After treatment, an electrophoresis test was performed via electrophoresis on 1.2% agarose gel with Tris–acetate (TAE) running buffer at 80 V for 25 min. DNA was visualized with EtB "Out" nucleic acid staining solution. The suspensions containing HAc-treated gene/vehicle complex, dextran sulfate-treated gene/vehicle complex, vehicle, gene/vehicle complex, naked gene and marker were respectively loaded in the loading wells of agarose gel. As shown, fluorescent signals from DNA molecules were not observed for the HAc-treated and dextran sulfate-treated gene/vehicle complexes. Gene/vehicle complex-HAc aggregation was not observed under visible light (not shown). Under UV light, no fluorescent signal was found. However, light aggregation of gene/vehicle complex-dextran sulfate was observed under visible light (not shown). Under UV light, the fluorescent signal of the aggregation contacting the dye-containing gel was observed in the region surrounding the bottom of loading well, implying that dextran sulfate demonstrated condensation ability for the proposed gene/vehicle complex. .
