# Supplementary figures and images for: An Engineered Gene Nanovehicle Developed for Smart Gene Therapy to Selectively Inhibit Smooth Muscle Cells: An In Vitro Study
Source: Int J Mol Sci. 2020 Feb 24;21(4):1530. doi: 10.3390/ijms21041530 (PMC7073206; doi:10.3390/ijms21041530)

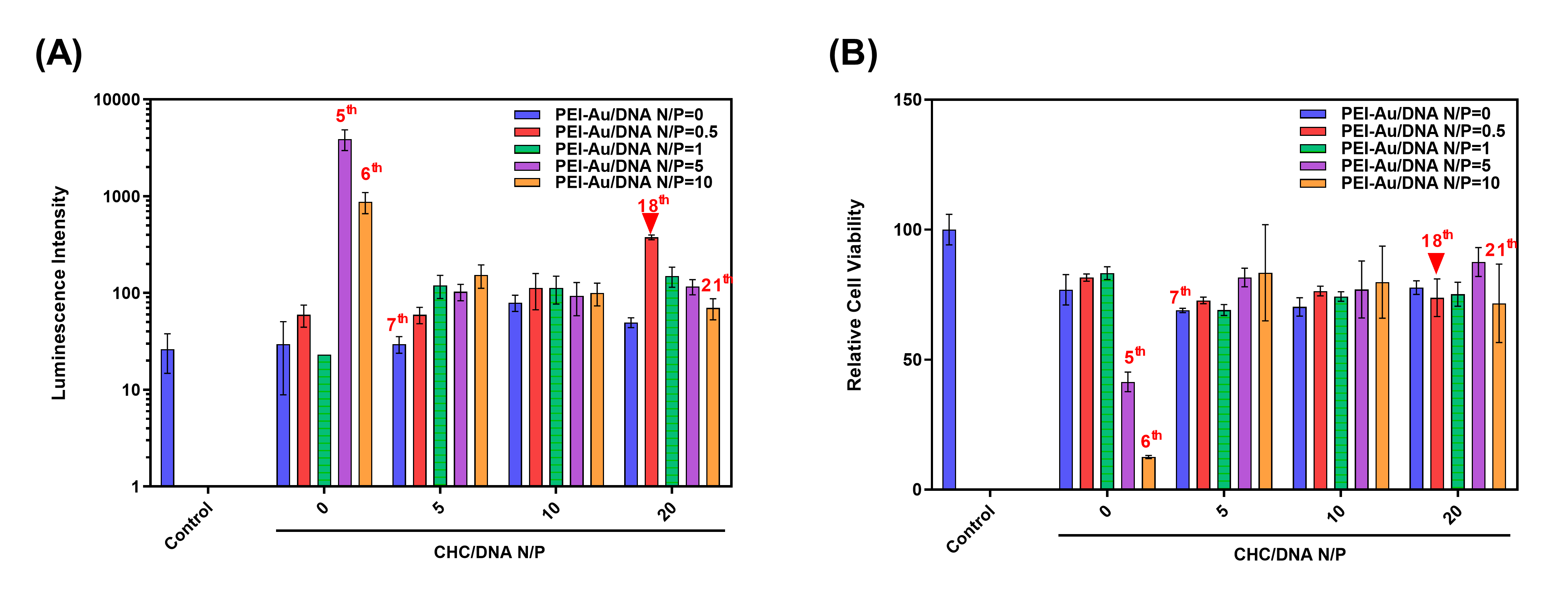

Supplement: Supplementary file 1 [file ijms-21-01530-s001.zip › Figure S1.tif]

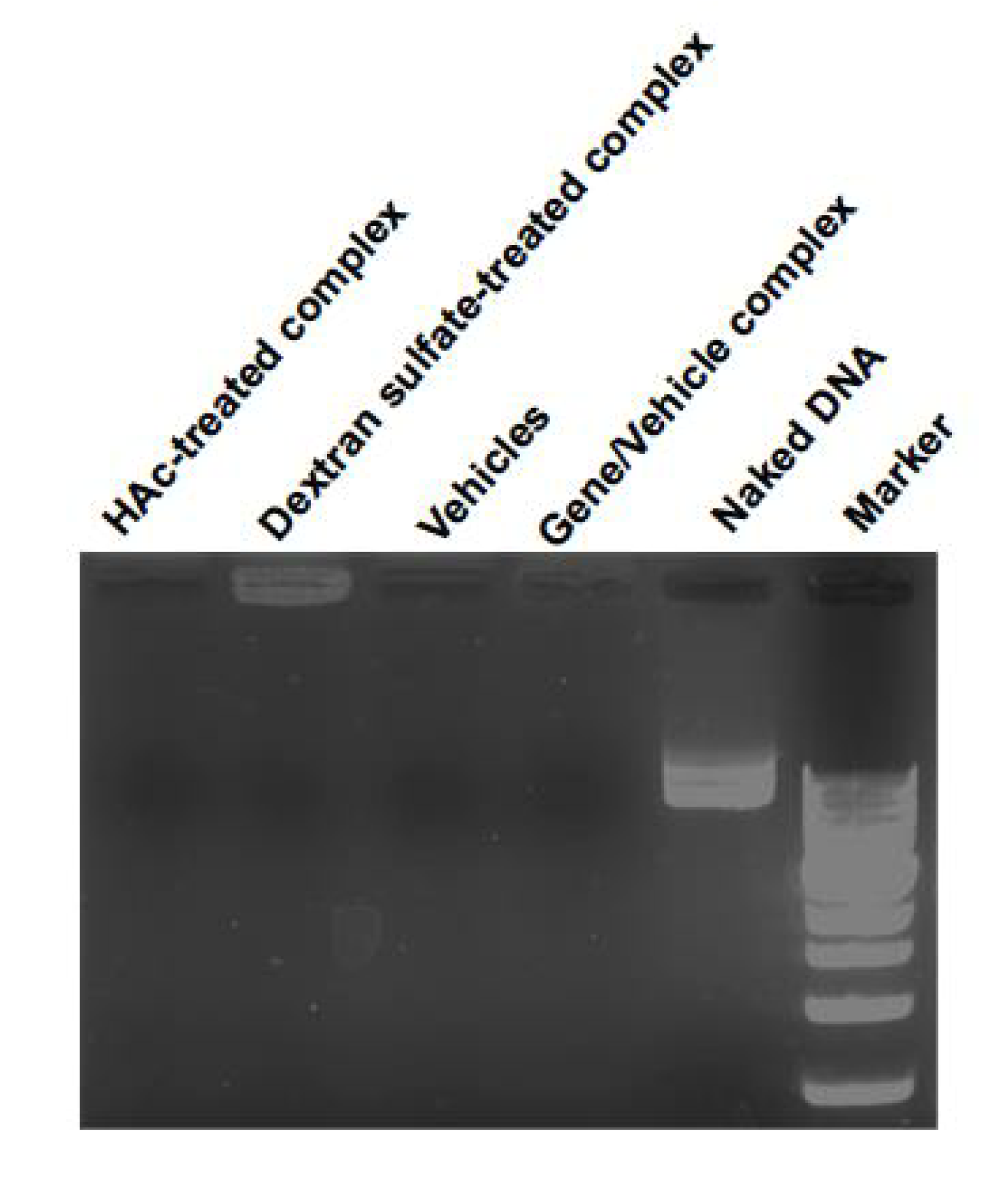

Supplement: Supplementary file 1 [file ijms-21-01530-s001.zip › Figure S2.tif]
